# Effect of Temperature, pH, and a_w_ on Cereulide Synthesis and Regulator Genes Transcription with Respect to *Bacillus cereus* Growth and Cereulide Production

**DOI:** 10.3390/toxins16010032

**Published:** 2024-01-08

**Authors:** Yating Wang, Yangtai Liu, Shuo Yang, Yuhang Chen, Yang Liu, Dasheng Lu, Hongmei Niu, Fanchong Ren, Anning Xu, Qingli Dong

**Affiliations:** 1 School of Health Science and Engineering, University of Shanghai for Science and Technology, Shanghai 200093, China; 213392838@st.usst.edu.cn (Y.W.); lyt@usst.edu.cn (Y.L.); shuoyang0117@163.com (S.Y.);; 2 Shanghai Center for Disease Control and Prevention, Shanghai 200336, China; 3 Key Laboratory of Milk and Dairy Products Detection and Monitoring Technology for State Market Regulation, Shanghai Institute of Quality Inspection and Technical Research, Shanghai 200233, China; liuyang@sqi.org.cn

**Keywords:** *Bacillus cereus*, cereulide, environmental factors, genes transcription, growth

## Abstract

*Bacillus cereus* is a food-borne pathogen that can produce cereulide in the growth period, which causes food poisoning symptoms. Due to its resistance to heat, extreme pH, and proteolytic enzymes, cereulide poses a serious threat to food safety. Temperature, pH, and a_w_ can influence cereulide production, but there is still a lack of research with multi-environmental impacts. In this study, the effects of temperature (15~45 °C), pH (5~8), and a_w_ (0.945~0.996) on the emetic reference strain *B. cereus* F4810/72 growth, cereulide production, relevant *ces* genes (*cesA*, *cesB*, *cesP*), and transcription regulators genes (*codY* and *abrB*) expression at transcription level were studied. *B. cereus* survived for 4~53 h or grew to 6.85~8.15 log_10_ CFU/mL in environmental combinations. Cereulide accumulation was higher in mid-temperature, acidic, or high a_w_ environments. Increased temperature resulted in a lower cereulide concentration at pH 8 or aw of 0.970. The lowest cereulide concentration was found at pH 6.5 with an increased a_w_ from 0.970 to 0.996. Water activity had a strong effect on transcriptional regulator genes as well as the *cesB* gene, and temperature was the main effect factor of *cesP* gene expression. Moreover, environmental factors also impact cereulide synthesis at transcriptional levels thereby altering the cereulide concentrations. The interaction of environmental factors may result in the survival of *B. cereus* without growth for a period. Gene expression is affected by environmental factors, and temperature and pH may be the main factors influencing the correlation between *B. cereus* growth and cereulide formation. This study contributed to an initial understanding of the intrinsic link between the impact of environmental factors and cereulide formation and provided valuable information for clarifying the mechanism of cereulide synthesis in combined environmental conditions.

## 1. Introduction

*Bacillus cereus* is a Gram-positive and facultative anaerobic foodborne pathogen that can pose a serious threat to food safety [1,2]. Approximately 1.4~12% of foodborne outbreaks worldwide are attributed to *B. cereus* [3]. In Europe, 413 foodborne outbreaks caused by *B. cereus* were reported between 2007 and 2014 [4]. Most outbreaks of foodborne illness caused by *B. cereus* have usually been associated with cell concentrations above 10^5^ CFU/g or CFU/mL in foods because *B. cereus* may produce cereulide (emetic toxin) when bacterial concentrations reach this level [4]. Cereulide is extremely stable in thermal, so food heating processing might inactivate the microorganism but would not destroy cereulide that is pre-formed in foods [5]. Cereulide can resist proteolytic enzymes in the gastrointestinal environment, be absorbed, and distributed throughout the body, causing severe multi-organ failure or even death after it is taken into the body [6,7]. Serious food poisoning outbreaks usually occur worldwide due to the high toxicity and stability of cereulide [8,9,10,11].

Cereulide ([D-O-Leu-D-Ala-L-O-Val-L-Val]3) is a cyclic dodecadepsipeptide (1.2 kDa) encoded by the *ces* cluster and produced by the activated non-ribosomal peptide synthetase (NRPS) [1]. The cereulide gene cluster comprises *cesPTABCD* operon and *cesH*. The *cesPTABCD* is transcribed as a polycistronic transcript from the main *ces* promoter located upstream of *cesP* and *cesH* is transcribed from its own promoter, which controls cereulide formation on a transcriptional level [12,13]. *CesP* encodes phosphotransferase to activate NRPS; the structural genes of *cesA* and *cesB* can integrate amino acid monomers into polypeptide chains which play an important role in the structure formation of cereulide [1,14]. Furthermore, cereulide-NRPS is the result of a complex and multi-layered process and is tightly regulated by the metabolite-responsive transcriptional regulators of AbrB, Spo0A, and CodY [13,15]. These regulators may promote or inhibit *ces* gene expression at the post-transcriptional level, thereby affecting the process of cereulide synthesis [16]. Environmental factors, such as temperature, pH value, and water activity (a_w_), are usually involved in food processing [17,18,19]. Currently, the temperature has been proven to have an impact on cereulide production and the expression of the cereulide synthetase gene [20,21]. Nevertheless, there is a notable scarcity of comprehensive investigations covering the (interactions) impact of pH and a_w_ on cereulide production [22,23,24]. These limitations hinder the ability to closely simulate real food production scenarios. Therefore, the aim of the paper was to monitor the *B. cereus* growth in combinations of temperature, pH, and a_w_ with cereulide production. Furthermore, an RT-qPCR assay was included to examine the influence of temperature, pH, and a_w_ on the expression of cereulide synthesis-related genes at a transcriptional level.

## 2. Results

### 2.1. Effect of Environments on Emetic B. cereus Behavior and Cereulide Production

#### 2.1.1. *B. cereus* Survival

**Figure 1:**
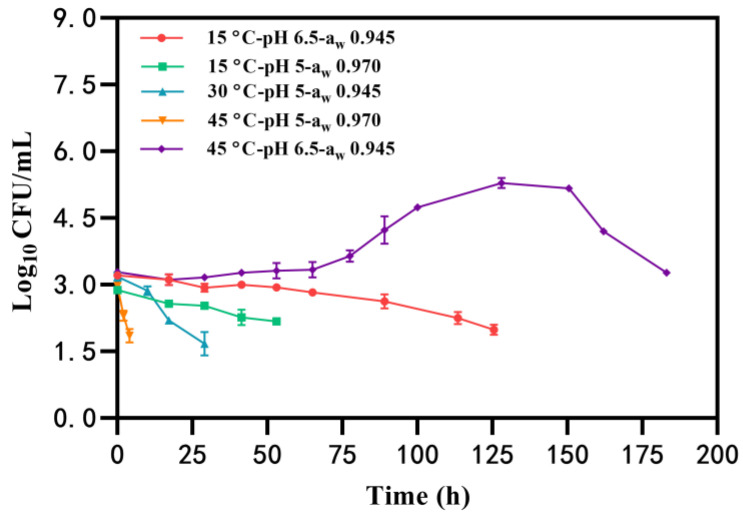
The survival of *B. cereus* in the condition of temperature, pH, and a_w_.

Under certain conditions, the growth of *B. cereus* was not observed, and the number of *B. cereus* counts gradually decreased to the detected limit (<50 CFU/mL) with an initial inoculation of 2~3 log_10_ CFU/mL (Table 1, Group I). Notably, *B. cereus* had the longest survival time (183 h) at the condition of 45 °C-pH 6.5-a_w_ 0.945, whereas the bacteria survived for the shortest time (approximately 4 h) at 45 °C-pH 5-a_w_ 0.970 (Figure 1). Similarly, the time that *B. cereus* was maintained without growth in BHI at pH of 5 and a_w_ of 0.970 (53 h) was less than that at pH 6.5-a_w_ 0.945 (125 h) at 15 °C (Figure 1). These findings suggest that the combination of pH 5-a_w_ 0.970 is unfavorable for *B. cereus* survival and that elevated temperatures may further reduce *B. cereus* counts in a shorter time.

#### 2.1.2. *B. cereus* Growth with or without Cereulide Production 

**Figure 2:**
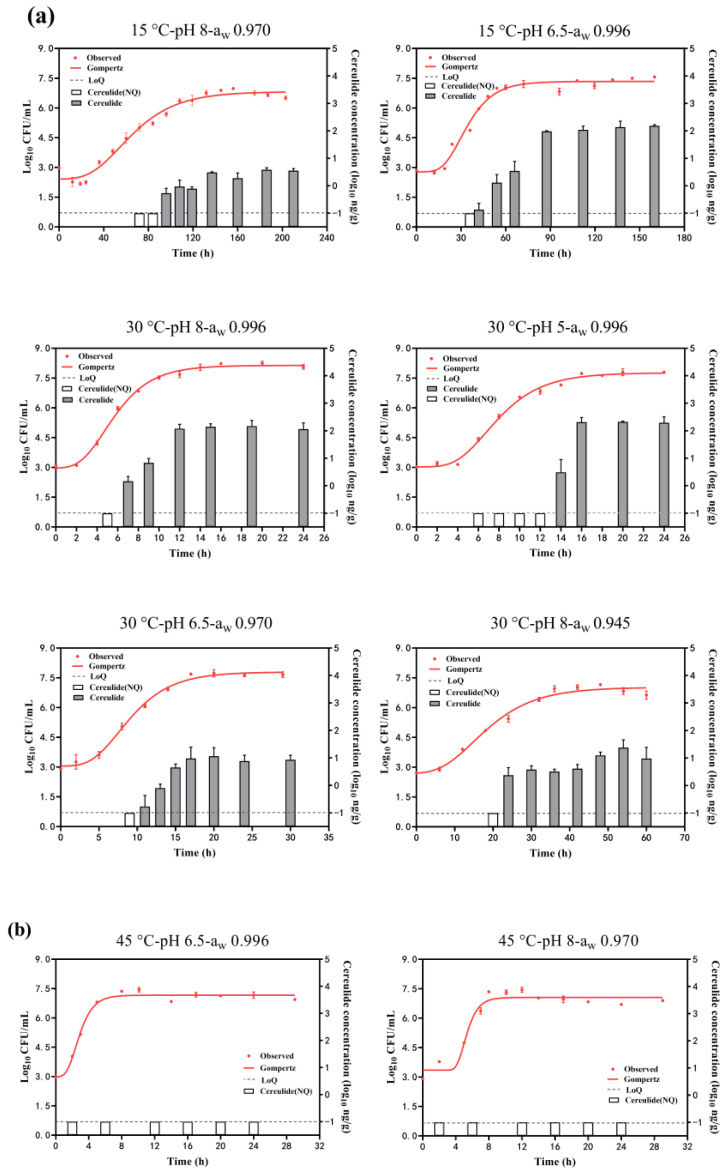
*B. cereus* growth and cereulide production in combinations range of temperature (15~45 °C), pH (5~8), and a_w_ (0.945~0.996). (**a**) growth with cereulide production; (**b**) growth without cereulide production. White columns mean the cereulide concentration (log_10_ ng/g) was lower than −1 log_10_ ng/g (dash lines, LoQ ng/g) (not quantitation) and gray columns mean the quantified cereulide.

Figure 2 shows the growth curves of *B. cereus* growth under different conditions by fitting the Gompertz model. The estimated parameters of the maximum population (*Y*_max_) obtained from it are summarized in Table 1 (Group II and Group III). The adj. R^2^ was in the range of 0.946~0.995 in each condition, showing the goodness-of-fit of this model. The *Y*_max_ was in the range of 6.85~8.15 log_10_ CFU/mL at the combination effects of temperature, pH, and a_w_. The highest *Y*_max_ was obtained at 30 °C, pH of 8, and a_w_ of 0.996, while the lowest *Y*_max_ was obtained at 15 °C, pH of 8, and a_w_ of 0.970. It was suggested that a decrease in temperature and a_w_ would result in a decrease in *Y*_max_ and would not favor *B. cereus* growth. The *Y*_max_ was around 7 log_10_ CFU/mL for other combinations with a slight difference. Meanwhile, as depicted in Figure 2a, it is evident that cereulide is usually produced in the mid to late exponential phase and gradually increases as *B. cereus* grows. The rate of cereulide production will gradually slow down or even tend to stabilize when *B. cereus* enters the growth stationary phase. However, no cereulide (<0.1 ng/g) was detected at the conditions combined with 45 °C, even though *B. cereus* grew to 7.05~7.16 log_10_ CFU/mL (Figure 2b). 

The concentrations of cereulide in the stationary phase of *B. cereus* growth under each condition are summarized in Table 1 (Group II). The maximum cereulide concentrations were 367 ng/g, observed at the condition of T:30 °C-pH 8-a_w_ 0.996, followed by 125 ng/g at the condition of T:30 °C-pH 5-a_w_ 0.996, and the minimum cereulide concentrations observed at the condition of T:15 °C-pH 8-aw 0.970 were just 3 ng/g. Cereulide concentrations were 119.16 ng/g at 15 °C, while no toxin was detected at 45 °C, indicating that higher temperatures were not conducive to cereulide production. Cereulide concentrations at pH 5 (78.05 ng/g) were higher than those at pH 8, suggesting that acidic conditions favored cereulide production. Similarly, cereulide concentrations at a_w_ 0.996 (130.95 ng/g) were higher than those at a_w_ 0.945, indicating that reduced a_w_ was disadvantageous for cereulide production.

In addition, both effects of factors can also be analyzed from Table 1. The condition of 15 °C-pH 6.5 reduced the cereulide concentration by 101.57 ng/g compared to the condition of 30 °C-pH 5 at a_w_ of 0.996, while the condition of 15 °C-pH 8 reduced the concentration by 7.04 ng/g compared to the condition of 30 °C-pH 6.5 at a_w_ of 0.970. It was indicated that cereulide concentration increased when the temperature increased with the pH decreased in the a_w_ range of 0.970~0.996. And decreased pH had a greater impact on cereulide concentration changes in acidic conditions. Cereulide concentrations increased by 139.53 ng/g when a_w_ increased from 0.970 to 0.996, while concentrations increased by 8.58 ng/g with a_w_ decreased from 0.970 to 0.945 when the temperature increased from 15 to 30 °C. And the lowest concentration was found at a_w_ 0.970. It was suggested that higher temperatures coupled with increased water activity resulted in higher cereulide concentrations. Furthermore, cereulide concentrations at pH 8-a_w_ 0.996 were 132.49 ng/g higher than at pH 6.5-a_w_ 0.970, and concentrations at pH 5-a_w_ 0.996 were 210.54 ng/g higher than at pH 6.5-a_w_ 0.970. It was indicated that the least favorable conditions for cereulide production were at pH 6.5 when a_w_ increased from 0.970 to 0.996, and that increased acidity led to higher cereulide concentrations.

### 2.2. Effect of Environments on Cereulide Synthesis-Related Gene Expression 

#### 2.2.1. Effect of a Single Factor 

**Figure 3:**
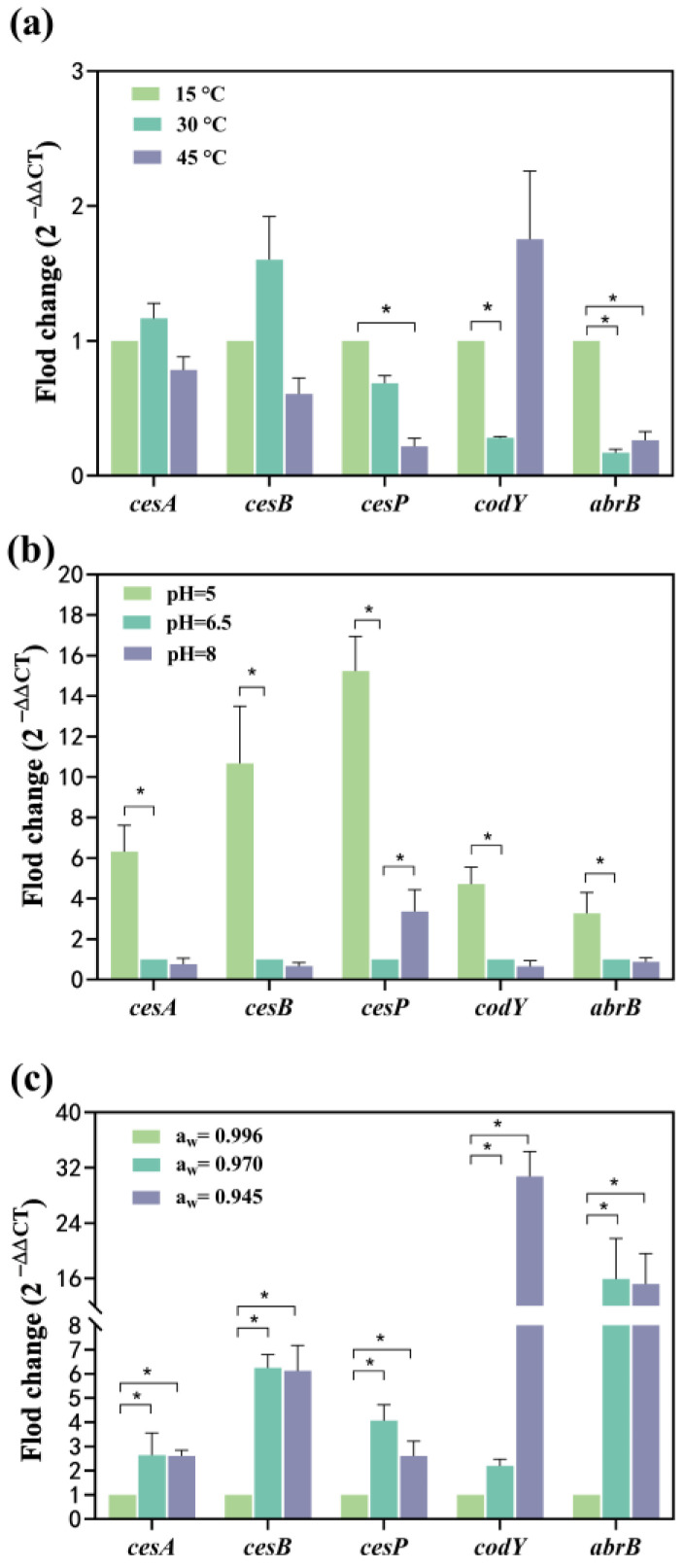
Effect of single factor on the transcription of *cesA*, *cesB*, *cesP*, *codY,* and *abrB* genes. (**a**) The temperature effect; (**b**) The pH effect; (**c**) The a_w_ effect. The fold change value for the control group was 1. 0 < 2^−ΔΔCT^ < 0.5 or 2^−ΔΔCT^ > 2, representing significant (*p* < 0.05) down- or up-regulation of genes, represented as *. Data represented as the mean ± standard deviations of three biological replicates.

Compared to 15 °C, the mRNA levels of *cesA* and *cesB* expression increased 0.16-fold and 0.6-fold at 30 °C, respectively, while they were down-regulated 0.21-fold and 0.39-fold at 45 °C, respectively (Figure 3a). This suggests a temperature-dependent modulation of *cesA* and *cesB* expression, initially enhanced and subsequently suppressed with increasing temperature. Conversely, the expression of the *cesP* gene showed a 0.32-fold reduction at 30 °C and a significant 0.78-fold reduction at 45 °C (Figure 3a), indicating suppression of gene expression with elevated temperatures. The transcriptional regulator genes, *codY* and *abrB*, were significantly down-regulated 0.72-fold and 0.83-fold at 30 °C, respectively. At 45 °C, *codY* was up-regulated 0.75-fold, while *abrB* was significantly down-regulated 0.73-fold. Both *codY* and *abrB* exhibited suppression when the temperature increased from 15 °C to 30 °C, with *abrB* showing a greater inhibition of gene expression. While the expression of the *codY* gene increased from 30 °C to 45° C, the *abrB* gene was less affected but still up-regulated (Figure 3a). This indicates that the mRNA expression of both genes decreased and then increased with the rising temperature from 15 to 45 °C, reaching the lowest expression at 30 °C.

Compared to the pH of 6.5, all tested genes exhibited significant up-regulation, ranging from 3.28- to 15.23-fold at pH 5. Notably, the relative mRNA levels expressed by *cesA*, *cesB*, and *cesP* (6.32–15.23-fold) surpassed those of *codY* and *abrB* (3.28~4.71-fold) (Figure 3b), and a lower pH environment was more conducive to the expression of *ces* genes. Among these, *cesP* demonstrated the most significant up-regulation (14.23 folds) within the *ces* gene cluster (Figure 3b). *CesA*, *cesB*, *codY*, and *abrB* genes experienced a 0.65–0.87-fold down-regulation, while *cesP* was significantly up-regulated (3.37-fold) at pH 8 (Figure 3b). This suggests that the pH range from 6.5 to 8 had a more substantial effect on *cesP* gene transcription, but other toxin synthesis-related genes were less affected. Furthermore, the mRNA levels expressed by *cesP* fluctuated with changes in pH, being lowest at a pH of 6.5, indicating that the acidic environment favored *cesP* gene expression.

Compared to a_w_ of 0.996, *cesA*, *cesB*, *cesP*, *codY*, and *abrB* were significantly up-regulated: 1.64-, 5.25-, 3.07-, 1.19-, and 14.92-fold at a_w_ 0.970, respectively, with the *abrB* exhibiting the highest mRNA level. At a_w_ of 0.945, all genes were significantly up-regulated 1.60-, 5.12-, 1.61-, 29.76-, and 14.21-fold, respectively, with the highest mRNA level observed for *codY* (Figure 3c). The mRNA expression levels of *cesA*, *cesB*, and *abrB* increased and then stabilized as a_w_ gradually decreased. The mRNA expression levels of *cesP* followed a pattern of increase followed by a decrease, peaking at a_w_ of 0.970. The a_w_ of 0.945 had the most pronounced effect on *codY* expression.

#### 2.2.2. Effects of Two Factors 

**Figure 4:**
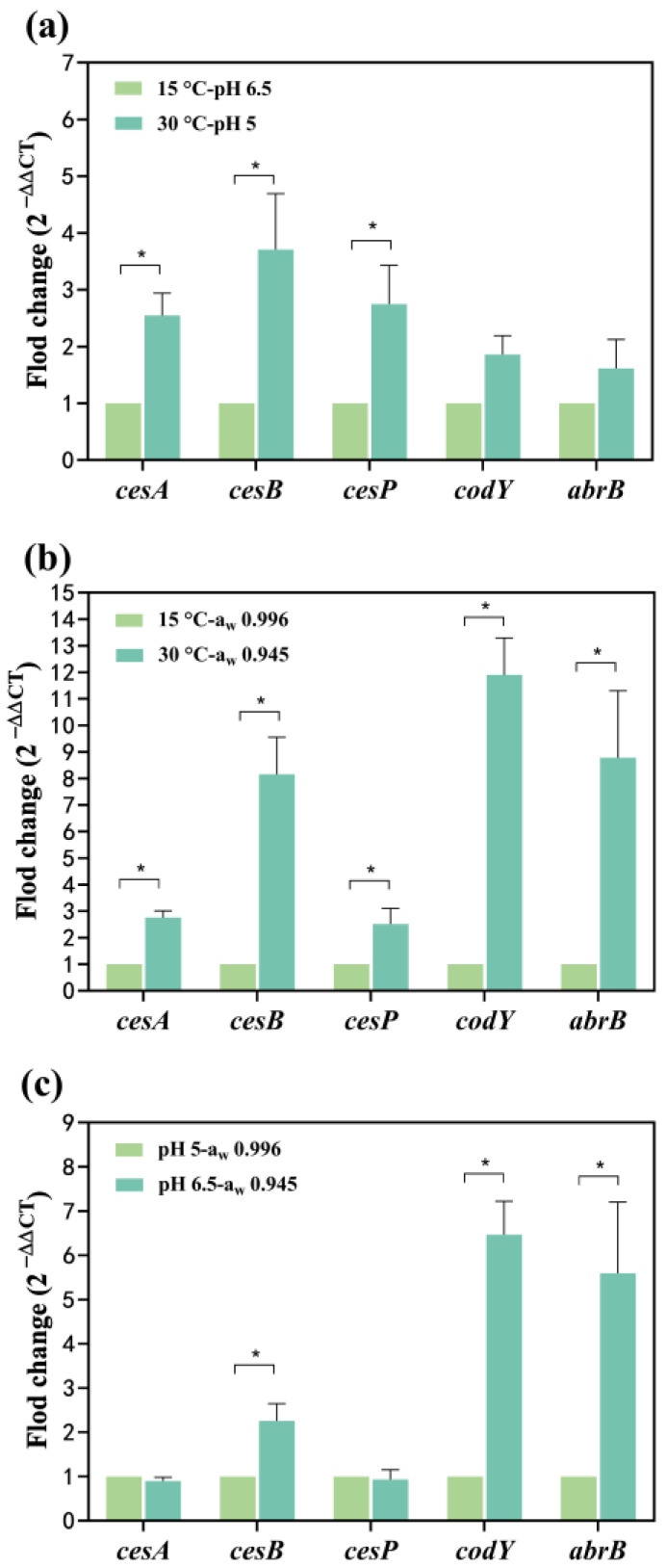
Effect of two factors on the transcription of *cesA*, *cesB*, *cesP*, *codY*, and *abrB* genes. (**a**) The temperature and pH effects; (**b**) The temperature and aw effects (**c**) The pH and a_w_ effects. The fold change value for the control group was 1. 0 < 2^−ΔΔCT^ < 0.5 or 2^−ΔΔCT^ > 2, representing significant (*p* < 0.05) down- or up-regulation of genes, represented as *. Data represented as the mean ± standard deviations of three biological replicates.

The *cesA* and *cesB* up-regulated 0.17 and 0.60-fold at 30 °C, respectively (Figure 3a). At pH 5, the *cesA* and *cesB* significantly up-regulated 5.32 and 9.67-fold, respectively (Figure 3b). Simultaneous exposure to temperature and pH conditions led to the up-regulation of *cesA* and *cesB*: 1.55 and 2.71-fold, respectively, at 30 °C-pH 5 compared to 15 °C-pH 6.5 (Figure 4a). The combined impact of temperature and pH on the mRNA expression levels of the *cesA* and *cesB* genes was intermediate between the individual effects, suggesting a greater combined effect of temperature and pH on these genes compared to either factor alone. The relative mRNA levels of the *cesP*, *codY*, and *abrB* (0.62~1.75-fold up-regulated) were higher at 30 °C-pH 5 than at 15 °C-pH 6.5 (Figure 4a). In addition, these genes were significantly up-regulated at pH 5 alone while down-regulated at 30 °C alone (Figure 3a,b), indicating that the enhancement of their expression due to reduced pH outweighed the inhibition caused by increased temperature.

When water activity and temperature acted simultaneously on *cesA* and *cesB* expression (Figure 4b), both genes up-regulated 1.75 and 7.15-fold at 30 °C-a_w_ 0.945, respectively. Notably, the combination of water activity and temperature resulted in a higher mRNA expression level for *cesB* compared to each factor alone, suggesting a synergistic promotion of gene expression. At 30 °C-a_w_ 0.945, *cesP*, *codY*, and *abrB* significantly up-regulated 1.52, 10.90, and 7.78-fold, respectively (Figure 4b). Comparatively, the expression of these genes increased when influenced by both water activity and temperature, indicating that the reduction in water activity had a more substantial impact on gene expression than the effect of increased temperature alone.

At pH 6.5-a_w_ 0.945, *cesB*, *codY*, and *abrB* significantly up-regulated 1.26, 5.47, and 4.59-fold, whereas the mRNA levels of the *cesA* and *cesP* genes were less changed compared to pH 5-a_w_ 0.996 (Figure 4c). Figure 3b illustrates that the pH effect alone significantly decreased transcription of all genes in the increased pH from 6.5 to 5. Conversely, a decrease in water activity (from 0.996 to 0.945) alone resulted in a significant up-regulation of all genes (Figure 3c). When both factors acted on the *cesA* and *cesP* genes, the inhibitory effect from the increase in pH may have neutralized the enhancement effect from the decrease in water activity, resulting in no significant change in the expression of both. For *cesB*, *codY*, and *abrB* expression, the impact of water activity may be greater than the effect of pH, resulting in the up-regulation of these genes.

## 3. Discussion

### 3.1. The Effect of Temperature, pH, and a_w_ on B. cereus Behavior and Cereulide Production

The emetic *B. cereus* F4810/72 strain exhibits growth across a temperature range of 15~45 °C, pH levels of 5~8, and a_w_ of 0.945~0.996 (NaCl concentrations of 0.5~8%) [25,26]. However, diverse environmental factors within this range may hinder *B. cereus* growth, allowing for substrate survival. Martínez et al. [27] demonstrated that *B. cereus* can endure without growth for 50 days in nutrient broth at 12–40 °C with a salt concentration exceeding 1% and a pH of 4.5. Similarly, in our result (Figure 1), *B. cereus* survived for 4~53 h at 15~45°C with the pH of 5~6.5 and a_w_ of 0.945~0.970 (corresponding salt concentration of 4.25~8%). Increased salt concentration may impede *B. cereus* survival due to reduced water activity, limiting water availability for bacterial metabolism and consequently reducing survival time [28]. Survival of *B. cereus* may have the potential for further growth [29] and even result in cereulide production if the counts are over 5 log_10_ CFU/mL (Figure 2). Therefore, minimizing *B. cereus* survival time under appropriate conditions is crucial to reduce the risk of growth and subsequent cereulide production.

In most conditions, the growth of *B. cereus* accompanied by cereulide production was observed across various temperature, pH, and a_w_ combinations (Figure 2a). The optimal condition for cereulide production was found at 30 °C, pH 5, and a_w_ 0.996 (Table 1), which is within the typical pH and water activity range of most foods. Therefore, prioritizing temperature control is essential to manage cereulide production. At 45 °C, temperature becomes dominant, potentially rendering cereulide undetectable (Figure 2b), aligning with previous studies [20,21]. Elevating the temperature to 45 °C was suggested to reduce the risk of cereulide production by *B. cereus.* Regarding the effect of pH on cereulide formation, our results indicated a higher cereulide production at pH 5 compared to pH 8 (Table 1). A similar phenomenon has been observed in acidic foods (pH 5.8) with higher concentrations of cereulide than alkaline foods (pH 7.9) [22]. However, this trend may not be universal for all cereulide-producing *B. cereus*. Guérin et al. [30] found that, when the pH decreased from 7 to 5.4, the cereulide concentration produced by *B. weihenstephanensis* (belonging to the *B. cereus* phylogenetic group) decreased. This may be due to the individual differences between different *B. cereus*. groups. In addition, our results indicated that cereulide concentrations decreased by 130.95 ng/g when the a_w_ was reduced from 0.996 to 0.945 (Table 1). Dommel et al. [24] also showed that the cereulide concentration decreased when the NaCl concentration increased. The increase in NaCl concentration led to a decrease in the available water in the environment and also adversely affected the *B. cereus* growth (Table 1). Therefore, cereulide produced in the growth phase may be inhibited in low a_w_ environments resulting in a reduction in cereulide concentration. Above all, mid-temperatures, acidic, or high a_w_ environments may result in higher cereulide concentrations. It is worth noting that temperature may act as a major factor influencing cereulide production at higher temperatures and the risk of cereulide formation may be lower at 45 °C.

In addition, we also explored the multifactorial situations that reduced cereulide concentration. The increased temperature with an increase in pH decreased cereulide concentration. Furthermore, the lowest concentration was found at an a_w_ of 0.970 at increased temperatures. When a_w_ increased from 0.970 to 0.996, a pH of 6.5 was the least favorable condition for cereulide production. These provided the reference for the environmental prevention and control of cereulide.

### 3.2. The Effect of Temperature, pH, and a_w_ on Cereulide-Related Gene Expression

The *ces* operon (*cesPTABCD*) controls cereulide formation and is expressed during *B. cereus* growth [13,30]. Environmental factors have been shown to influence gene expression at the transcriptional level. The *cesA* and *cesB* are structural genes, encoding the cereulide NRPS, which are responsible for the D-Ala-D-O-Leu fragment and L-Val-L-O-Val fragment, respectively [12]. The *cesA* and *cesB* were often significantly up-regulated by the decreased pH (from 6.5~5) or a_w_ (0.996 to 0.945) (Figure 3b,c), indicating that these conditions were favorable for the formation of D-Ala-D-O-Leu and L-Val-L-O-Val fragments. Dommel et al. [24] found that the relative expression of the *cesA* decreased with decreasing a_w_ during the exponential growth phase of *B. cereus*, whereas the present study found that the relative expression of this gene increased with decreasing in a_w_ in the stationary growth phase, suggesting that the expression of *cesA* may not be consistently suppressed by increasing salt concentration during the *B. cereus* growth cycle. In addition, we found that the maximum relative expression of *cesA* and *cesB* occurred at 30 °C, which might be the optimal temperature for the formation of cereulide structures, which was consistent with the trend of results from Kranzler et al. [21]. The *cesP* gene encodes a phosphopantetheinyl transferase to activate NRPS [1]. And the *cesP*_1_ promoter is located upstream of *cesP* and drives the transcription of the *cesPTABCD* operon [22]. Currently, changes in the expression of this gene as influenced by environmental factors are unknown. Our study found that the increased temperature from 15 °C to 45 °C or pH of 6.5 was detrimental to *cesP* expression (Figure 3a,b), thereby inhibiting the synthesis of the phosphotransferase and affecting the transcription of *ces* operon. While a_w_ of 0.970 may have a positive effect on the cereulide biosynthesis process due to the transcription level of *cesP* being enhanced. Therefore, reduced pH or a_w_ favored the formation of the cereulide structure, and the most optimal temperature was 30 °C. pH and a_w_ had opposite trends on *cesP* expression, and increased temperature was detrimental to NRPS activation.

Transcriptional regulators are also involved in the process of *ces* expression [12]. CodY, a global transcriptional regulator, is linked to the metabolic state and virulence expression of the bacterial cell [31]. In *B. cereus*, CodY acts as a repressor of the *ces* operon, thereby diminishing the cereulide formation [32]. Another transcriptional regulator, AbrB, negatively influences cereulide synthesis by binding to the promoter of the *cesP* gene and inhibiting its expression [15]. The mRNA levels of *codY* and *abrB* were at their lowest at 30 °C (Figure 3a), thereby reducing their inhibitory impact on cereulide biosynthesis at the transcriptional level. In contrast, the expression of *codY* and *abrB* was significantly up-regulated in environments with pH 5 or a_w_ 0.945 (Figure 3b,c), suggesting that these conditions are conducive to the synthesis of CodY and AbrB. Consequently, their heightened presence enhances the negative regulation of the cereulide formation process. Therefore, the negative effect of transcription factors on cereulide synthesis was minimal at 30 °C. While the negative regulation increased progressively with decreasing pH or a_w_, thus unfavorably affecting cereulide formation. 

Furthermore, the impact of temperature and pH, or temperature and a_w_, on the expression of *cesA* and *cesB* does not appear to follow a single factor. There may be interactive effects between these factors, giving rise to either antagonistic or synergistic effects on gene expression. Consequently, the mRNA expression levels may deviate from a mere summation of individual effects. Notably, the influence of water activity on *cesB* expression surpassed that of pH, whereas both factors exhibited similar effects on the expression of *cesA* and *cesP*. Temperature emerged as a pivotal factor governing *cesP* expression. Interestingly, water activity had the greatest effect on the expression of transcriptional regulator genes of *codY* and *abrB*, followed by pH, with temperature exerting the least effect.

### 3.3. Relationship between B. cereus Growth and Cereulide Production

The observed lack of strict correlation between cereulide formation and *B. cereus* growth may be attributed to extrinsic environmental factors playing a direct role in the expression of cereulide synthetase genes, which agreed with the findings by Kranzler et al. [21] and Dommel et al. [30]. In our study, the *Y*_max_ of *B. cereus* could reach 7 log_10_ CFU/mL at 45 °C, which was not much different from that at 15 °C. Despite this, cereulide was not detected, possibly due to the down-regulation of *cesA*, *cesB*, and *cesP* genes at 45 °C, as illustrated in Figure 3a. This down-regulation inhibits cereulide synthesis by affecting both the formation of the cereulide structure and the activation of cereulide synthase. Meanwhile, the substantial up-regulation of the *codY* may increase the inhibitory effect of the transcription factor CodY on the process of cereulide synthesis. In addition, the effect of the negative regulation of *abrB* on cereulide production may be less than that of other genes because no toxins were detected at 45 °C. Besides temperature, pH also contributed to the differences in *B. cereus* growth and cereulide formation. Table 1 shows that a pH value of 5 was unfavorable for *B. cereus* growth but favorable for cereulide production. The promotion of cereulide synthesis, as evidenced by higher relative mRNA levels of *cesA*, *cesB*, and *cesP* compared to *codY* and *abrB*, likely contributed to the increase in final cereulide concentration under the former condition. Above all, temperature and pH are important factors that may affect the correlation between *B. cereus* growth and cereulide formation.

To the best of our knowledge, this study represents the inaugural investigation into cereulide production under the influence of diverse environmental factors and its consequential impact on *B. cereus* gene expression at the transcriptional level. Gaining insight into the influence of extracellular factors on cereulide synthesis is crucial for delineating inhibitory conditions, thereby facilitating the formulation of appropriate measures for preventing and controlling cereulide. Additionally, this paper analyzed the pattern of gene expression associated with cereulide synthesis in response to environmental factors, providing an initial understanding of the mechanism governing cereulide synthesis in combined environmental conditions. Nonetheless, the pathways through which *B. cereus* cells perceive environmental signals and the mechanisms orchestrating the integration of these signals into physiological metabolism to activate the cereulide biosynthesis process remain unclear. Further elucidation of these aspects would contribute to a deeper understanding of the cereulide biosynthesis pathway in *B. cereus* under environmental influences.

## 4. Conclusions

*B. cereus* exhibits the capability to endure without active growth in the culture medium over a specific duration, contingent upon the interplay of temperature, pH, and water activity (a_w_). The association between *B. cereus* growth and cereulide production is noteworthy; however, a precise correlation may not always exist. This divergence may be attributed to the direct influence of environmental conditions on the transcriptional activity of *B. cereus* cereulide-related genes, potentially resulting in variations in cereulide concentration. It is imperative to recognize that diverse environmental factors exert distinct impacts on gene expression, and the degree of influence varies among different genes. Notably, temperature emerges as a key determinant governing *cesP* gene expression. Furthermore, water activity significantly influences the expression of transcriptional regulator genes during cereulide synthesis, with temperature exhibiting a comparatively lesser impact. The interaction of these environmental factors and their potential effects on gene expression adds complexity to our understanding of the regulatory mechanisms underlying *B. cereus* behavior in specific conditions.

## 5. Materials and Methods

### 5.1. Preparation of Stock Culture

The standard emetic strain of *B. cereus* F4810/72 was used in this study (purchased from DSMZ, Braunschweig, Germany). The strain stored in 50% glycerin at −80 °C was inoculated in 10 mL Brain Heart Infusion (BHI) broth (Hopebiol, Qingdao, China) and incubated for 20 h at 30 °C. Then, the culture was enumerated on Tryptone Soya Agar (TSA) plates (Hopebiol, Qingdao, China) and incubated for 20 h at 30 °C. One isolated colony was inoculated into BHI broth and incubated for 18 h at 30 °C to have a working culture. To prove the absence of spores, the culture was heated at 80 °C for 10 min and spread on TSA plates. After overnight incubation, there was no colony growth, and no spores were observed in the culture by a microscope, indicating that there were no spores in the initial bacterial culture [33].

### 5.2. Experimental Design

The experimental ranges were determined according to the single-factor growth range of emetic *B. cereus* strain F4810/72, three common factors in food processing, temperature of 15~45 °C, pH of 5~8, and a_w_ of 0.996~0.945 (conversed from 0.5~8% NaCl) were selected to explore the growth of *B. cereus* under multiple factors [24,25], and the experimental groups were designed by Box–Behnken design [34]. Additional conditions of 30 °C-pH 6.5-a_w_ 0.996 and 30 °C-pH 6.5-a_w_ 0.945 were supplemented in gene transcription results.

### 5.3. Samples Inoculation and Growth Quantification

A conical flask (200 mL volume) containing 120 mL of BHI broth was adjusted to the desired a_w_ and pH with NaCl and HCl/NaOH, respectively. The conversion relationship between NaCl concentration and a_w_ was referenced in the study of Carlin et al. [22]. BHI broth (200 mL volume) was supplemented with the desired NaCl so that the NaCl concentration of the final solution was 0.5%, 4.2%, and 8% (*w*/*v*). The a_w_ of 0.945, 0.970, and 0.996 correspond to NaCl concentrations of 8%, 4.2%, and 0.5% (*w*/*v*), respectively. Then, it was inoculated with an overnight culture of *B. cereus* F4810/72 to reach an initial target concentration of about 10^2^~10^3^ CFU/mL vegetative cells. The inoculum was incubated in tested conditions. Samples were taken at intervals depending on the preliminary experiments to ensure at least 1 to 2 observations in each logarithmic level of the bacterial population during the growth of *B. cereus*. Test samples were serially diluted 10-fold in saline and 60 µL were transferred for drop and spread plating three times on TSA plates and then incubated for colony counting. Three countable drops were used to calculate the average of the total number of colonies [35]. Three biologically independent replicates were performed for each condition. 

### 5.4. Growth Modelling

The growth curves of *B. cereus* under different conditions were fitted by the *Gompertz* model using the GraphPad Prism version 8.3 (La Jolla, CA, USA, 2019), presented in Equation (1) [36]. The maximum population (*Y*_max_) was obtained from the bacterial growth curves to compare the ability to grow in different environmental conditions. If the increase in *B. cereus* counts did not exceed 2 log_10_ CFU/mL, the growth was considered not significant, and no fit was applied [20]. The adjusted coefficient of determination (Adj. R^2^) was used to evaluate the goodness-of-fit of the model [37].

(1) $$Y\left(t\right)=Y_{0}+\left(Y_{max}-Y_{0}\right)exp\left\{-exp\left[\frac{2.17\mu_{max}\left(\lambda -t\right)}{Y_{max}-Y_{0}}+1\right]\right\}$$

where *Y*(*t*) is the bacterial population at *t* in ln CFU/mL, *Y*_0_ is the initial population in ln CFU/mL, *μ*_max_ is the maximum specific growth rate (1/h), *Y*_max_ is the maximum population in ln CFU/mL, and *λ* is the lag time in h.

### 5.5. Cereulide Extraction

The experimental conditions for cereulide extraction were in accordance with bacterial growth conditions. Interval extraction of the cereulide during the *B. cereus* exponential growth phase in each condition. The *B. cereus* cell pellets were obtained from the 120 mL bacterial culture after centrifugation at 8600× *g* for 10 min. The ^13^C_6_-cereulide (Chiralix, Nijmegen, The Netherlands) was added to the pellets as internal standard and then equilibrated for 30 min at 25 °C. Each sample was extracted by shaking with acetonitrile on a rocking table for 18 h at 20 °C. The extract was centrifuged at 8600× *g* for 15 min, the supernatant obtained was centrifuged at 11,000× *g* for 8 min, then membrane filtered (0.2 μm, PTFE membrane) to remove cell debris [38,39].

### 5.6. Cereulide Quantification 

The analysis was performed on a Shimadzu Nexera X2 LC-30AD UPLC system coupled with a Shimadzu LCMS-8060 mass spectrometer (Kyoto, Japan). The samples from cell extractions were injected into the 1.7 μm 50 × 2.1 mm column (Waters, Milford, MA, USA) at 40 °C. The isocratic elution was made with 90% acetonitrile-0.1% (*v*/*v*) formic acid (solvent A) and 10% 10 mM ammonium formate-0.1% (*v*/*v*) formic acid solution (solvent B) and the flow rate was 0.4 mL/min [18]. The MS-MS analysis was carried out using a Shimadzu LCMS-8060 triple quadrupole with an electrospray ionization (ESI) interface. The instrument was operated in positive mode in the multiple reaction monitoring (MRM) mode. The collision energy for the cereulide was set to 55 eV. The transitions (*m*/*z*) used for obtaining the daughter fragments of cereulide were 1170.7 → 314.4, 1170.7 → 499.4. The collision energy for the ^13^C_6_-cereulide was set to 90 and 74 eV, and the transitions (*m*/*z*) used for obtaining the daughter fragments of ^13^C_6_-cereulide were 1176.7 → 172.2, 1176.7 → 315.4 [39].

The limit of quantification (LoQ) was defined based on a S/N ratio > 10. The LoQ in this study was determined at 0.1 ng/g in the tested bacterial cell pellets. The recovery rates were 103%, 97%, and 92% in the cereulide concentrations were 0.02, 0.15, and 5 ng/mL, respectively (Table S1). The standard calibration curve was established by cereulide (Chiralix, Nijmegen, The Netherlands) and ^13^C_6_-cereulide (internal standard) (Figure S1). The concentration of cereulide is expressed in log_10_ ng/g (nanogram per gram of wet cell pellet). 

### 5.7. Quantitative RT-PCR

Three genes of the *ces* gene cluster (*cesA*, *cesB*, and *cesP*) and two transcriptional regulator genes (*abrB* and *codY*) were selected from the cereulide biosynthesis process. A 16S rRNA gene (*rrn*) was used as an internal reference. The primer sequences were presented in Table S2. *B. cereus* cells were collected from the growth stabilization phase. The cultures were centrifuged at 10,000× *g* for 2 min at 4 °C and the pellets were immediately put on ice. Total RNA was extracted from the cells using a Bacteria Total RNA Isolation Kit (Genstone Biotech, Beijing, China). cDNA was obtained by reverse transcription using the first-strand cDNA synthesis kit (Vazyme Biotech Co., Ltd., Nanjing, China), according to the instructions of manufacturer. 

Quantitative real-time PCR (RT-qPCR) was carried out in a 20 μL reaction volume containing 10 μL SYBR^®^ Green Master Mix (Vazyme Biotech Co., Ltd., Nanjing, China), 0.4 μL of each primer (10 μmol/mL), 0.5 μL of DNA template and 8.7 μL PCR-grade water. The PCR temperature profile was 95 °C for 30s, followed by 40 cycles of 95 °C for 10 s, and 60 °C for 30 s. After the final cycle, samples were incubated for a further 15 s at 95 °C, 60 s at 60 °C, and 15 s at 95 °C. Relative gene expression (fold change) was calculated by the 2^−ΔΔCT^ method [40,41].

## Figures and Tables

**Table 1:** The maximum population and cereulide concentration of *B. cereus* at combinations of temperature, pH, and a_w_.

Group	Temperature (°C)	pH	a_w_	*Y*_max_ (log_10_ CFU/mL)	Cereulide Concentration (ng/g) ^1^	Adj. R^2^
I. No growth	15	6.5	0.945	NG	-	-
	15	5	0.970	NG	-	-
	30	5	0.945	NG	-	-
	45	5	0.970	NG	-	-
	45	6.5	0.945	NG	-	-
II. Growth with cereulide production	15	8	0.970	6.85 ± 0.04	3.15 ± 0.81	0.975
	15	6.5	0.996	7.30 ± 0.07	119.16 ± 22.21	0.978
	30	8	0.996	8.15 ± 0.06	142.68 ± 13.82	0.995
	30	5	0.996	7.76 ± 0.07	220.73 ± 11.77	0.992
	30	6.5	0.970	7.10 ± 0.07	10.19 ± 2.26	0.988
	30	8	0.945	7.02 ± 0.06	11.73 ± 5.14	0.978
III. Growth without cereulide production	45	6.5	0.996	7.16 ± 0.07	<0.1 ^2^	0.983
	45	8	0.970	7.05 ± 0.06	<0.1 ^2^	0.946

^1^ Data represented means ± standard deviations from at the latest three time points during *B. cereus* growth stabilization phase; ^2^ Cereulide concentration was lower than LoQ; NG, no growth; -, no result; Values are means ± standard deviations of three biological duplicate experiments.

## Data Availability

The data presented in this study are available on request from the first author.

## Supplementary Materials

The following supporting information can be downloaded at: https://www.mdpi.com/article/10.3390/toxins16010032/s1, Figure S1: The standard curve of quantifying cereulide standard solution used ^13^C_6_-cereulide as internal standard. (a) The concentrations of 0.02~1 ng/mL (0.15 ng/mL); (b) 1~15 ng/mL (5 ng/mL); Table S1: The recovery rate and limit of quantification (LOQ) of cereulide; Table S2: Primers developed for RT-qPCR. References [15,24,42] are cited in the supplementary materials.
