# Supplementary material for: Effect of Temperature, pH, and aw on Cereulide Synthesis and Regulator Genes Transcription with Respect to Bacillus cereus Growth and Cereulide Production
Source: Toxins (Basel). 2024 Jan 8;16(1):32. doi: 10.3390/toxins16010032 (PMC10818934; doi:10.3390/toxins16010032)
Supplement: Supplementary file 1 [file toxins-16-00032-s001.zip › toxins-2774350-supplementary.pdf]

Effect of temperature, pH, and a_w on cereulide synthesis and regulator genes transcription with respect to *Bacillus cereus* growth and cereulide production

Yating Wang, Yangtai Liu, Shuo Yang, Yuhang Chen, Yang Liu, Dasheng Lu, Hongmei Niu and Qingli Dong

As shown in Fig. 1, the synthetic cereulide was dissolved in acetonitrile to concentrations of 0.02, 0.05, 0.1, 0.2, 0.5, and 1 ng/mL; 1, 2, 4, 7, 10 and 5 ng/mL with the concentrations of synthetic $^{13}C_6$-cereulide (internal standard) was 0.15 ng/mL and 5 ng/mL, respectively. These standard curves were used for the quantification of cereulide at low and high concentrations, respectively.

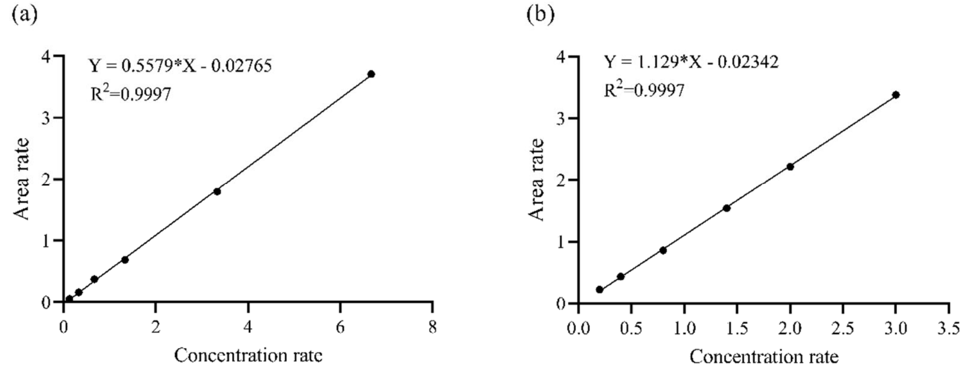

Figure S1 The standard curve of quantifying cereulide standard solution used $^{13}C_6$-cereulide as internal standard. (a) The concentrations of 0.02~1 ng/mL (0.15 ng/mL); (b) 1~15 ng/mL (5 ng/mL).

Table S1. The recovery rate and limit of quantification (LoQ) of cereulide.

Cereulide concentration (ng/mL)	Recovery rate (%)	LoQ* (ng/g)
0.02	103 (±7.4)	
0.15	97 (±5.6)	0.10
5	92 (±1.6)	

* Limit of quantification, S/N>10; Data represented as the mean ± standard deviations of three replicates.

Table S2. Primers developed for RT-qPCR**.**

Primer	Sequence (5′-3′)	Reference
16SrRNA (*rrn*)	F: GGAGGAAGGTGGGGATGACG<br>R: ATGGTGTGACGGGCGGTGTG	Dommel et al. [1]
cesA	F: GATTACGTTCGATTATTTGAAG<br>R: CGTAGTGGCAATTTCGCAT	Dommel et al. [1]
cesB	F: TTAGATGGTATTCTTCACTTGGC<br>R: TTGATACAAATCGCATTCTTATAACC	Dommel et al. [1]
cesP	F: GGTTATGCATCTTGTATACCG<br>R: GATGAAGTGGAGATGATATAGAC	Dommel et al. [1]
codY	F: CCACGACGGCTAACTACGAA<br>R: GCGTTATTACAGAGCGCAGC	Li et al. [2]
abrB	F: TCGTGTAGTAATTCCGATTGAA<br>R: TGAAGCTCGTTTAAGATTTGC	Lucking et al. [3]

References

1. Dommel, M.K.; Lücking, G.; Scherer, S.; Ehling-Schulz, M. Transcriptional kinetic analyses of cereulide synthetase genes with respect to growth, sporulation and emetic toxin production in *Bacillus cereus*. *Food Microbiol.* **2011**, *28*, 284–290. https://doi.org/10.1016/j.fm.2010.07.001.
2. Li, Y.; Chen, N.; Wu, Q.; Liang, X.; Yuan, X.; Zhu, Z.; Zheng, Y.; Yu, S.; Chen, M.; Zhang, J. A flagella hook coding gene *flgE* positively affects biofilm formation and cereulide production in emetic *Bacillus cereus*. *Front. Microbiol.* **2022**, *13*, 897836. https://doi.org/10.3389/fmicb.2022.897836.
3. Lucking, G.; Dommel, M.K.; Scherer, S.; Fouet, A.; Ehling-Schulz, M. Cereulide synthesis in emetic *Bacillus cereus* is controlled by the transition state regulator AbrB, but not by the virulence regulator PlcR. *Microbiology* **2009**, *155*, 922–931. https://doi.org/10.1099/mic.0.024125-0.
